# The Efficacy and Safety of B-Cell Maturation Antigen (BCMA) Antibody-Drug Conjugates (ADC) in Development against Cancer: A Systematic Review

**DOI:** 10.32604/or.2025.070851

**Published:** 2025-12-30

**Authors:** Jing Shan, Catherine King, Harunor Rashid, Veysel Kayser

**Affiliations:** 1 School of Pharmacy, The University of Sydney, Sydney, NSW 2006, Australia; 2 Sydney School of Public Health, The University of Sydney, Sydney, NSW 2006, Australia; 3 Sydney Infectious Diseases Institute, The University of Sydney, Westmead, NSW 2145, Australia; 4 Clinical School, The Children’s Hospital at Westmead, Westmead, NSW 2145, Australia

**Keywords:** B-cell maturation antigen, antibody drug conjugates, multiple myeloma, belantamab mafodotin, ocular toxicity, clinical trials

## Abstract

**Objectives:**

B-cell maturation antigen (BCMA)-targeted antibody–drug conjugates (ADCs) have emerged as promising therapies for relapsed/refractory multiple myeloma (RRMM), but the overall efficacy and safety profile is unclear. This study aimed to synthesize the available evidence on the safety and efficacy of BCMA-ADCs in development for RRMM.

**Methods:**

A systematic search was conducted using six bibliographic databases and ClinicalTrials.gov up to November 2024. Studies were eligible if they were human clinical trials or animal studies evaluating BCMA-ADCs and reported efficacy and safety outcomes. Data extraction and quality assessments were conducted using validated tools, including ROBINS-I and SYRCLE’s risk of bias tool.

**Results:**

A total of 21 studies were included: 16 clinical trials and five animal studies. Key findings included that belantamab mafodotin demonstrated variable but generally durable response rates (32%–85%) and a broad range of progression-free survival (PFS) (2.8–36.6 months), albeit with ocular toxicities in 51%–96%. Among newer candidates, MEDI2228 showed median PFS 5.1–6.6 months with 14% discontinuation for ocular symptoms, while AMG 224 had an overall response rate (ORR) of 23% (9/40) with anemia 21%, thrombocytopenia 24%, and ocular adverse events (AEs) 21%. Animal studies supported the tumor-eradicating potential of all BCMA-ADC candidates, although safety signals such as hepatic and renal toxicity were noted with HDP-101. The risk of bias assessment revealed generally moderate to serious concerns in human trials, while the overall quality of the animal studies was acceptable.

**Conclusions:**

BCMA-targeted ADC candidates show encouraging efficacy in RRMM, particularly belantamab mafodotin. However, frequent AEs, especially ocular and hematologic toxicities, underscore the need for optimization in ADC design. Further research should prioritize enhancing safety while maintaining clinical benefit.

## 1 Introduction

Cancer is one of the major threats to human health, with large individual and societal impacts. According to a Global Cancer Observatory report, the estimated number of new cancer cases in 2022 was around 20 million, and almost 10 million of these had fatal outcomes [1]. Additionally, the burden of new cases was projected to rise to 28.4 million by 2024 [2]. Efficient and customized cancer therapies are urgently required. Cancer therapies can employ either non-specific methods or specific methods. The non-specific methods are surgery, chemotherapy, and radiotherapy, which not only destroy the cancer cells but also cause damage to healthy tissues [3,4]. In contrast, specific therapies comprise molecularly targeted agents, monoclonal antibodies (mAbs) and antibody drug conjugates (ADC), and immune approaches such as checkpoint inhibitors, chimeric antigen receptor T-cell therapy (CAR-T), and bispecific T-cell engagers, which aim to cause cytotoxicity in malignant cells while sparing normal tissues [5–7].

**Figure 1:**
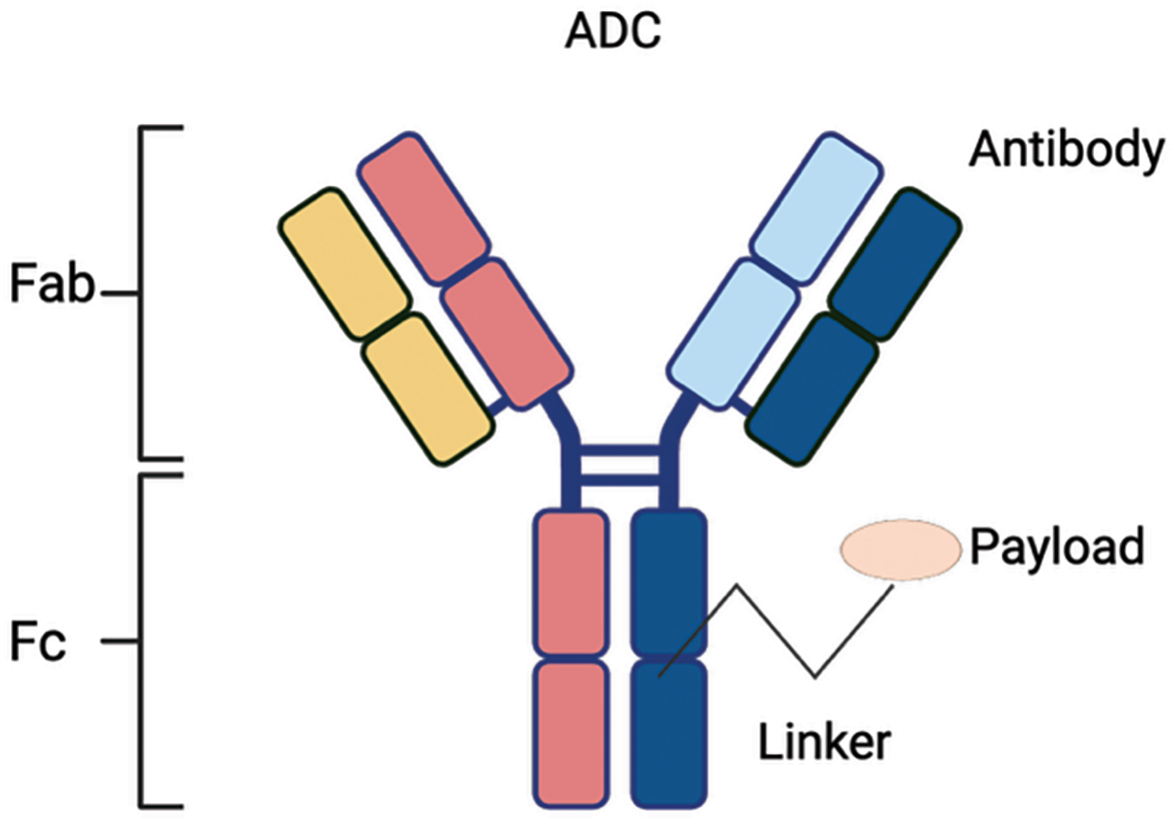
ADC structure: A target-specific monoclonal antibody is chemically linked to a cytotoxic payload via a linker. Fab, Fragment antigen-binding; Fc, Fragment crystallizable. Created via DBA BioRender (Science Suite Inc., Toronto, ON, Canada)

ADC is one of the specific treatment methods, which comprises a monoclonal antibody and a cytotoxic drug (payload) conjugated via a linker. The characteristics of an ideal ADC are based on a mAb with a high specificity for targeted tumor antigen, a blood circulation stable linker only cleavable in the target site, and a payload inducing cancer cell death after internalization and release (Fig. 1) [8].

B-cell maturation antigen (BCMA) is a transmembrane protein that is considered to be a member of the tumor necrosis receptor superfamily [9]. It can promote the normal B-cell differentiation and the growth and survival of malignant myeloma cells, due to the binding to the B-cell activating factor and a proliferation-inducing ligand [9,10]. BCMA is well-suited for ADC development because it shows high, relatively selective surface expression on late B-lineage/plasma cells with minimal expression in most normal tissues, limiting on-target/off-tumor effects. As a single-pass transmembrane receptor, BCMA internalizes after antibody binding to traffic payloads to endo-lysosomal compartments; although some antigen is shed as soluble BCMA, membrane density can be pharmacologically increased (e.g., γ-secretase inhibition) to enhance target engagement [11–13]. Currently, only one BCMA-ADC named belantamab mafodotin (Blenrep^®^) has been authorized by the US Food and Drug Administration (FDA) for treating patients with multiple myeloma, but clinical trials for other BCMA ADCs are still ongoing [14]. Additionally, other hematological malignancies also express BCMA, including B-cell lymphoblastic leukemia and lymphomas [15]. Therefore, BCMA could be used as a potential target for other hematological malignancies. To our knowledge, there is no systematic review synthesizing the efficacy and safety data of all BCMA-ADCs in development.

## 2 Methods

### 2.1 Study Object

The aim of our review is to systematically summarize the efficacy and safety data from BCMA-ADC human clinical trials and animal studies.

This systematic review was prospectively registered in the International Prospective Register of Systematic Reviews (PROSPERO): CRD42022349971 (https://www.crd.york.ac.uk/prospero/display_record.php?ID=CRD42022349971, accessed on 01 October 2025).

### 2.2 Literature Search

Searches were undertaken by an experienced medical information specialist (CK) in the following databases: Ovid Medline (1946 to 15 November 2024), Ovid Embase Classic (1947 to 1973); Embase (1974 to 15 November 2024), the Cochrane Library Database of Systematic Reviews (Issue 11 of 12, 2024) and Cochrane Central Register of Controlled Trials (Issue 10 of 12, 2024), SCOPUS (1823–November 2024), and Web of Science Core Collection (Science Citation Index Expanded 1900–November 2024, Social Sciences Citation Index 1956–November 2024, Arts & Humanities Citation Index 1975–November 2024, Conference Proceedings Citation Index–Science 1990–November 2024, Conference Proceedings Citation Index–Social Science & Humanities 1990–November 2024, Book Citation Index–Science 2005–November 2024, Book Citation Index—Social Sciences & Humanities 2005–November 2024, Emerging Sources Citation Index 2005–November 2024, Current Chemical Reactions 1985–November 2024, Index Chemicus 1993–November 2024). Conceptually, the search included (BCMA/CD269/TNFRSF17 terms) AND (ADC terms) AND (efficacy/effectiveness/safety terms). No language or date limits were applied. The full search strategy used and PRISMA checklist are available in Supplementary material S5 [16]. The final search was conducted on 20 November 2024. ClinicalTrials.gov was also searched on 20 November 2024 to identify ongoing or completed trials.

### 2.3 Screening

Items retrieved by the search were screened against the inclusion and exclusion criteria by the first author and cross-checked by two senior authors. A study was included in this systematic review if: (a) the study related directly to the use of BCMA-ADCs, (b) included human or animal study data, c) contained data from a clinical trial or observational study, (c) had one or more outcomes with efficacy and safety data such as, response, progression-free survival (PFS), and adverse events (AE). Studies involving participants of any age group and published in any language were considered. The items were excluded if they did not provide efficacy or safety data or if there was insufficient data for extraction.

### 2.4 Data Extraction

Two data extraction forms were developed by the first author in consultation with co-authors: one for human studies and the other for animal studies. For the human studies the following data were extracted: year of publication, study type, inclusion criteria, methodology, aim, country, study duration, follow-up, diagnosis, high risk of disease, extramedullary disease, stage of disease, sample size and patient characteristics, ethnicity, gender, age, BCMA-ADC, mAb, drug, linker, dosage, treatment duration, prior lines of therapy, sample size of different prior lines, duration of response (DOR), overall response, partial response (PR), very good partial response (VGPR), complete response (CR), minimal response (MR), median PFS, rate of survival, median overall survival, estimated 1-year survival, serious treatment related AEs, non-serious treatment related AEs, number of patients with treatment delay, number of patients with dose reduction, number of patients with treatment discontinuation, and any limitations acknowledged by the authors [17]. For the animal studies, year of publication, country, cancer cell lines, generation of animal model, types of patient-derived xenograft model, sample size, BCMA-ADC, dosage, survival rate, tumor-free days, side effects, drug antibody ratio, aim, delivery, pharmacokinetics/pharmacodynamics data, organs affected by ADCs, sex of the animal, mAb, drug, linker, and conjugation were extracted.

### 2.5 Data Synthesis and Quality Assessment

The first author summarized the efficacy of BCMA-ADCs in humans based on the months of duration of response, median PFS, and median overall survival; the rates of overall response and estimated 1-year survival; and the patient numbers of VGPR, PR, and CR. We also summarized the efficacy of animal studies based on the survival rate data and the number of tumor-free days. Moreover, the first author summarized the safety in humans via the treatment related serious AEs (SAEs)/non-serious AEs and the number of patients with treatment delay, discontinuation, and dose reduction. Quality assessments were conducted using the risk of bias in non-randomized studies - of interventions (ROBINS-I) tool for the human studies [18] and the Systematic Review Center for Laboratory Animal Experimentation (SYRCLE)’s risk of bias tool for animal studies [19]. Additionally, a quality assessment tool introduced by Chou et al. was used for studies reporting AEs, which is based on eight criteria. Each study was scored either 0 (inadequate) or 1 (adequate) for each criterion. Studies without enough information for an accurate assessment of a criterion were rated as inadequate. The overall quality ratings were as follows: good => 6, fair = 4–6, poor =< 4 [20].

## 3 Result

**Figure 2:**
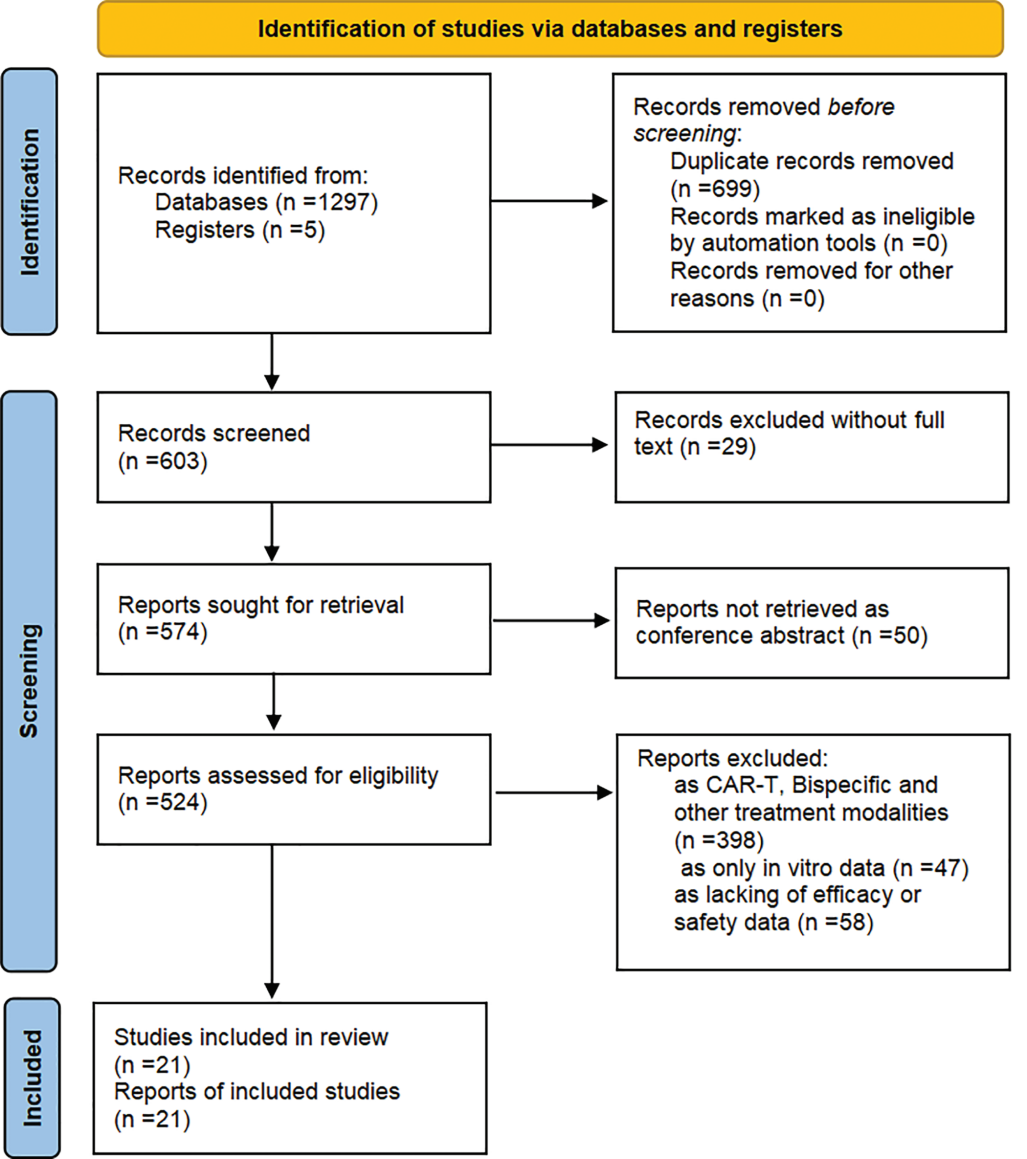
PRISMA flow chart

A total of 1302 records were identified. Of these, 699 were duplicates. Twenty nine were without full text, and another 50 were conference abstracts. A further 503 records were excluded following title and abstract screening, as they did not meet the inclusion criteria. Finally, 21 studies were included following full text review: 16 human studies and five animal studies (see Fig. 2 for PRISMA flow chart).

### 3.1 Clinical Studies

Fourteen of 16 human studies were related to belantamab mafodotin, and the other two were related to MEDI2228 and AMG 224 against multiple myeloma (see Tables 1, S1 and S3). A comprehensive summary of efficacy and safety outcomes across clinical studies has been compiled and presented in Table 1 to improve interpretability and support cross-study comparisons.

**Table 1:** The efficacy and safety in the clinical trials

BCMA ADC	Study type	Patient number and gender	Overall response	Median of progression-free survival (months)	Rate of survival	Treatment related to serious adverse events	Author
Belantamab-mafodotin	Phase 1/2 trial (DREAMM-8)	46 males and 41 females	85.30%	21.8	52.8% in 2 years	Grade ≥3 keratopathy (52.6%), decreased visual acuity (39.5%), thrombocytopenia (34.2%), neutropenia (36.8%)	Trudel et al. [21]
MEDI2228	Phase 1 (MEDI2228)	73 were males and 34 were females	39.3% (95% CI: 30.0%–49.2%)	3.4	NA	Photophobia (43.9%), thrombocytopenia (19.6%)	Dimopoulos et al. [22]
Belantamab-mafodotin	Phase 1/2 (DREAMM-6)	35 males and 10 females	67% (95% CI: 51.0%–80.0)	NA	NA	1 related to treatment	Popat et al. [23]
Belantamab-mafodotin	Phase 1/2 (DREAMM-4)	19 males (56%) and 15 females (44%)	16	3.4	85% at 12 months and 73% at 18 months	12% of patients (4 cases)	Suvannasankha et al. [24]
Belantamab-mafodotin	Phase 3 (DREAMM-3)	184 males and 141 females	41% (95% CI: 34.2%–47.7%)	11.2	48% (95% CI: 40–56%) in 12 months	Thrombocytopenia: 23% (49/217 patients) and anemia: 16% (35/217 patients)	Dimopoulos et al. [25]
Belantamab-mafodotin	Retrospective observational study	55 males and 47 females	37%	5.5	NA	Grade 3 keratopathy (2.5%), Grade 3 thrombocytopenia (9%)	Fazio et al. [26]
Belantamab-mafodotin	Phase 1/2 BelaRd study	19 males and 17 females	83.30%	NA	NA	Grade ≥3 fatigue (58.3%), rash (16.7%), keratopathy (18.1%)	Terpos et al. [27]
Belantamab-mafodotin	Retrospective multicenter study (Spanish Expanded Access Program)	72 males and 84 females	41.80%	3.61	NA	Corneal events (33.7%), thrombocytopenia (15.4%)	de la Rubia et al. [28]
Belantamab-mafodotin	Phase 2 trial (DREAMM-2)	NA	32% (2.5 mg/kg cohort), 35% (3.4 mg/kg cohort)	2.8 (2.5 mg/kg cohort), 3.9 (3.4 mg/kg cohort)	87.4% at 2 years	Grade ≥3 keratopathy (52.6%), neutropenia (36.8%), thrombocytopenia (34.2%)	Nooka et al. [29]
Belantamab-mafodotin	Retrospective study	25 males and 11 females	29%	2 (95% CI 1.4–2.7)	40%–50%	Grade 3 AEs = 5 (14%). All were keratopathies.	Abeykoon et al. [30]
Belantamab-mafodotin	Observational, retrospective, multicenter study	15 males and 18 females	42.20%	3 (95% CI: 0.9–5.1)	NA	12 patients (36.4%) had severe TEAEs, including keratopathy (51.5%) and thrombocytopenia (21.2%)	Alegre et al. [31]
AMG 224	Phase 1 study (AMG 224) NCT02561962	19 males and 21 females	23% (95% CI: 11–39%)	NA	NA	Grade 4 thrombocytopenia (24%), anemia (21%), and ocular AEs in 21% of patients	Lee et al. [32]
Belantamab-mafodotin	One-arm study (Phase 2, DREAMM-2)	51 males and 46 females	32%	Overall: 2.8 (95% CI 1.6–3.6); VGRs: median PFS 14 (95% CI 7.5–NR); PRs: median PFS 6.2 (95% CI 2.8–NR); CRs: median PFS NR (95% CI 7.1–NR)	42/95	Grade ≥3 AEs = 54 (57%) and 1 was a fatal AE. 11 in the HR-IMWG; 19 in the HR-Cyto	Lonial et al. [33]
Belantamab-mafodotin (refrigerated lyophilized powder presentation)	One-arm study (Pivotal, Phase 2, DREAMM-2, NCT03525678)	14 males and 11 females	52%	5.7 (95% CI, 2.2–9.7)	15/25	Treatment-related SAEs = 4 (17%). The most common were keratopathies.	Richardson et al. [34]
Belantamab-mafodotin	One-arm study (Phase 1) NCT02064387	17 males and 18 females	60%	12.0 (95% CI 3.1–NR); refractory to IMiDs & PIs: median PFS 7.9 months (95% CI 2.3–NR); no prior daratumumab: median PFS 15.7 months (95% CI 2.3–NR); prior daratumumab: median PFS 6.8 months (95% CI 1.3–NR); prior daratumumab + refractory to IMiDs & PIs: median PFS 6.2 months (95% CI 0.7–7.9).	31/35	Treatment-related SAEs = 7 (20%).	Trudel et al. [35]
Belantamab-mafodotin	One-arm study (Phase 1); Part 1 for dose selection, Part 2 for safety and efficacy NCT02064387	Part 1: 20 were male and 18 were female. Part 2: 17 were male and 18 were female	60%	Part 2: 7.9 (95% CI 3.1–NR)	NA	Part 2: Treatment-related SAEs = 5 (14%). Infusion-related reactions (n = 2), intracranial hemorrhage (n = 1), lung infection and pyrexia (n = 1), and pericardial effusion (n = 1).	Trudel et al. [36]

Note: BCMA, B-cell maturation antigen; ADC, antibody-drug conjugate; ORR, Overall Response Rate; PFS, Progression-Free Survival; CI, Confidence Interval; CR, Complete Response; PR, Partial Response; VGR, Very Good Response; HR-IMWG, High-Risk by International Myeloma Working Group criteria; HR-Cyto, High-Risk Cytogenetics; AE, Adverse Event; SAE, Serious Adverse Event; TEAE, Treatment-Emergent Adverse Event; NR, Not Reached; EAP, Expanded Access Program; Phase 1/2, Combined Phase 1 and 2 Clinical Trial; NA, Not Available; IMiDs, Immunomodulatory Imide Drugs.

Of the included human clinical studies, 11 were multinational trials, while the remaining studies were conducted in individual countries, including 2 in the USA and 1 each in Italy, Greece, and Spain.

Belantamab mafodotin is conjugated with a BCMA IgG1 antibody and the microtubule polymerisation inhibitor monomethyl auristatin F (MMAF) via a protease-resistant maleimidocaproyl (mc) linker. In 2018, Trudel et al. published the results of a phase I trial, the one-arm DREAMM-1 study (NCT 02064387), about the safety and tolerability of belantamab mafodotin among relapsed/refractory multiple myeloma (RRMM) patients. This was a dose escalation and expansion trial including two parts. In the beginning, 38 patients were recruited who were aged around 60 years. The administered dose was 0.03–4.60 mg/kg. No dose limiting toxicities and maximum tolerated dose were identified. Subsequently, another 35 patients, with a median age of 60 years, were recruited who were administered 3.4 mg/kg belantamab mafodotin. The median PFS was 12 months. The overall response rate (ORR) was 60%, and the 25th percentile of DOR was 6.7 months. Five of the 35 patients had treatment-related SAEs. Of these, 25 of 35 patients had treatment delay, and 23 of 35 patients had dose reduction, which were due to blurred vision and thrombocytopenia. Another two patients had to discontinue treatment due to thrombocytopenia and increased serum creatinine phosphokinase [36]. In 2019, Trudel et al. also published the updated results of the dose expansion part of the previous phase I trial after an additional 14-month follow-up. Thirty-one of 35 patients survived following treatment with belantamab mafodotin 3.4 mg/kg. The commonest Grade ≥3 AEs included thrombocytopenia (35%), anemia (17%), and keratopathy (14%), while ocular toxicities (69%) were the primary safety concern, leading to dose reductions in 46% of patients. Four other patients died due to the progression of multiple myeloma. There was no treatment-related death [35].

In 2020, Richardson et al. [34] reported findings from a phase II trial, the pivotal DREAMM-2 study (NCT03525678), about the efficacy and safety of the lyophilized form of belantamab mafodotin in an independent, exploratory cohort of patients who were followed up for a median of 11.2 months. A total of 25 patients were recruited, all had RRMM, and 24 received belantamab mafodotin at 3.4 mg/kg every three days. The ORR was 52, with the VGPR being 24%. The median response duration was 9 months, and the median PFS was 5.7 months. The most common treatment-related SAE was keratopathy, with a rate of 17%. Overall, the clinical response and safety profile of this lyophilized form of belantamab mafodotin were acceptable [34].

Nooka et al. [29] published the findings of the DREAMM-2 Phase II trial (NCT03525678) in 2023, which evaluated single-agent belantamab mafodotin in heavily pretreated RRMM patients who were triple-class refractory (resistant to a proteasome inhibitor (PI), an immunomodulatory drug, and an anti-CD38 monoclonal antibody). Conducted across multiple global sites in the United States, Canada, Europe, and Australia, the study included 223 patients receiving either 2.5 mg/kg (n = 97) or 3.4 mg/kg (n = 99) of belantamab mafodotin every 3 weeks, along with a lyophilized cohort (n = 25, 3.4 mg/kg dose). The patients were followed up for a median of 12.5 to 13.8 months; the ORR was 32% (2.5 mg/kg) and 35% (3.4 mg/kg), with higher responses (52%) in the lyophilized cohort. For the 2.5 mg/kg group the median PFS time was 2.8 months (95% CI: 1.6–3.6) and for the 3.4 mg/kg group the median PFS was 3.9 months (95% CI: 2.0–5.8), while median overall survival was 15.3 months (95% CI: 9.9–18.9) and 14.0 months (95% CI: 10.0–18.1), respectively. The median DOR was 12.5 months (2.5 mg/kg) and 6.2 months (3.4 mg/kg). Minimal residual disease (MRD) negativity was achieved in 36% (2.5 mg/kg) and 23% (3.4 mg/kg) of patients with ≥VGPR. Ocular toxicity remained a key safety concern, with keratopathy affecting 71% (2.5 mg/kg), 75% (3.4 mg/kg), and 96% (lyophilized cohort) of patients, while Grade ≥3 thrombocytopenia (38%–57%) and anemia (27%–28%) were also reported. Despite frequent dose modifications (54%–79% requiring dose delays, 36%–58% requiring reductions, and 12% discontinuing treatment due to AEs), the safety profile remained manageable, with no new safety signals identified. Patient-reported outcomes suggested stable or improved quality of life (QoL), with reductions in pain and fatigue. This final analysis confirms that belantamab mafodotin delivers durable responses in triple-class refractory RRMM patients, reinforcing its clinical potential despite ocular toxicity concerns, which require careful management and dose modifications [29].

In 2023, the DREAMM-3 Phase III trial (NCT04162210), led by Dimopoulos et al. [25], aimed to assess the safety and efficacy of belantamab mafodotin alone vs. pomalidomide combined with low-dose dexamethasone among RRMM patients. Conducted across 18 countries with 325 patients (median age 68 years), the study randomized participants 2:1 to either receive belantamab mafodotin (2.5 mg/kg IV every 21 days) or pomalidomide (4 mg daily, days 1–21) plus dexamethasone (40 mg weekly) in 28-day cycles. The primary endpoint, PFS, showed numerical but not statistically significant improvement with belantamab mafodotin (11.2 vs. 7.0 months, *p* = 0.56). Similarly, the DOR was not reached, and overall survival was comparable (21.2 vs. 21.1 months, *p* = 0.75). The ORR was 41% vs. 36%, with a higher very good partial response rate (25% vs. 8%). The commonest Grade 3–4 AEs in the belantamab mafodotin group were thrombocytopenia (23%) and anemia (16%), whereas pomalidomide-dexamethasone was associated with neutropenia (33%) and anemia (18%). Ocular toxicity (blurred vision 40%, keratopathy 12%) was a major safety concern, leading to 71% requiring dose modifications. No treatment-related deaths occurred in the belantamab mafodotin group, highlighting the need for combination strategies to enhance its efficacy in RRMM treatment [25].

In 2024, Suvannasankha et al. [24], published the DREAMM-4 Phase I/II trial (NCT03848845) outcomes. It evaluated the safety and efficacy of belantamab mafodotin plus pembrolizumab among RRMM patients. Conducted at 11 sites across the United States, Canada, Germany, and Spain, the study enrolled 41 patients (6 in Part 1, 28 in Part 2) who had received ≥3 prior lines of treatment, namely, an immunomodulatory drug, a PI, and an anti-CD38 mAb. Patients received belantamab mafodotin (2.5 or 3.4 mg/kg in Part 1; 2.5 mg/kg in Part 2) in combination with pembrolizumab (200 mg IV every 3 weeks) for a maximum of 35 cycles. They were followed up for a median of 18.9 months. The ORR was 47%, with 29% achieving a VGPR or better, including four CRs. MRD negativity was achieved in three of 10 patients with ≥VGPR, and among eight patients with prior anti-BCMA therapy, 62.5% (5/8) achieved a PR or better, including two patients who were refractory to anti-BCMA therapy. The median DOR was 8.0 months (95% CI: 2.1–not reached (NR)), the median PFS was 3.4 months (95% CI: 1.4–5.6), and the median overall survival was not reached (95% CI: 19.1–NR), with 12-month and 18-month overall survival rates of 85% and 73%, respectively. The commonest Grade ≥3 AEs were keratopathy (38%), thrombocytopenia (29%), and anemia (21%), while blurred vision (38%), infusion-related reactions (32%), pyrexia (32%), and nausea (29%) were also frequently reported. Dose reductions and interruptions were required in 32% and 65% of patients, respectively, but there were no treatment-related discontinuations. Despite no new safety signals, ocular toxicity remained a major concern, necessitating frequent dose modifications. While the combination demonstrated clinical activity, it did not show a substantial durability advantage over belantamab monotherapy (DREAMM-2 study), leading to no further planned investigations of this combination in RRMM [24].

In 2024, the DREAMM-6 Phase I/II trial (NCT03544281), published by Popat et al. [23], aimed to evaluate the efficacy and safety of belantamab mafodotin along with lenalidomide plus dexamethasone among RRMM patients who were treated with ≥1 prior line of therapy. The trial included a dose-escalation and dose-expansion phase, testing four belantamab mafodotin dosing regimens: 1.9 and 2.5 mg/kg every 4 weeks (SINGLE cohort), 2.5 mg/kg split between days 1 and 8 (SPLIT cohort), and 1.9 mg/kg every 8 weeks (STRETCH cohort). Across 45 patients (median age 68, range 36–80, 78% male), with a median follow-up of 23.7 months, ORR was 67% (95% CI: 51.0–80.0), with 29% achieving CR and 47% achieving VGPR or better. MRD negativity was observed in 22.2% of patients with ≥VGPR. The median PFS was 18.4 months (95% CI: 6.8–NR), with PFS not reached in the 1.9 mg/kg STRETCH and 2.5 mg/kg SINGLE cohorts. No dose limiting toxicities were reported, but AEs [17] were observed in all patients, with Grade ≥3 keratopathy (53%), neutropenia (22%), thrombocytopenia (22%), and reduced visual acuity (22%) being most common. Ocular AEs [17] occurred in 80% of patients, with 69% being Grade ≥3, primarily keratopathy (78%), reduced visual acuity (33%), and blurred vision (31%). Median time to first Grade ≥2 ocular event ranged from 29.0 to 41.5 days, with 76% of events resolving after a median of 85.5 days (range: 22–680 days). SAEs were recorded in 53% of participants, with four fatal SAEs, including one treatment-related febrile neutropenia case in the 2.5 mg/kg SINGLE cohort. Despite the high ocular toxicity rates, belantamab mafodotin plus lenalidomide and dexamethasone demonstrated clinically meaningful anti-myeloma activity, with manageable safety and comparable efficacy across dosing regimens. The 1.9 mg/kg STRETCH regimen showed the fastest resolution of ocular events while maintaining efficacy, suggesting potential benefits for dose modification strategies [23].

Also in 2024, the DREAMM-7 Phase III trial (NCT04246047) was published by Hungria et al. [37], which aimed to compare the efficacy and safety of belantamab versus daratumumab, both combined with bortezomib and dexamethasone, among RRMM patients who were treated with at least one prior line of therapy. Conducted across multiple global sites, the trial enrolled 494 patients (median age 65 years, 53% male) in a 1:1 randomization to receive either belantamab mafodotin or daratumumab. With a median follow-up of 28.2 months, median PFS was 36.6 months (95% CI: 28.4–NR) in the belantamab mafodotin group vs. 13.4 months (95% CI: 11.1–17.5) in the daratumumab group, demonstrating a significant PFS advantage for belantamab mafodotin. At 18 months, overall survival rates were 84% (belantamab mafodotin) vs. 73% (daratumumab), though the overall survival data were not mature at the time of analysis. The ORR was 83% (95% CI: 77–87) with belantamab mafodotin vs. 71% (95% CI: 65–77) with daratumumab, and CR or better was achieved by 35% vs. 17% of patients, respectively. MRD negativity was seen in 25% of belantamab mafodotin patients vs. 10% of daratumumab patients. However, Grade ≥3 AEs were higher with belantamab mafodotin (95%) than with daratumumab (78%), with ocular toxicity a major concern (79% vs. 29%), requiring dose modifications in 44%, delays in 78%, and discontinuation in 9%. Despite higher ocular toxicity, belantamab mafodotin significantly improved PFS and response rates, positioning it as a promising alternative regimen for RRMM patients [37].

The DREAMM-8 Phase I/II trial (NCT03715478) was published by Trudel et al. in 2024 [21]. The researchers evaluated the efficacy and safety of belantamab mafodotin plus pomalidomide and dexamethasone in RRMM patients who were lenalidomide-refractory and PI-exposed. It enrolled 87 patients (median of three prior treatment regimens, 55.2% triple-class refractory) in a dose-exploration (n = 61) and dose-expansion (n = 26) phase, with 38 patients receiving at the 2.5 mg/kg belantamab mafodotin every 8 weeks (Q8W) plus pomalidomide and dexamethasone. The ORR was 85.3% at RP2D (87.6% in all patients), with 75.7% achieving VGPR or better, 33.3% achieving CR or better, and MRD negativity in 5 of 7 assessed patients (3 of 4 at RP2D). The median PFS was not reached at RP2D (21.8 months in all patients), with an estimated 2-year PFS of 52.8%, while median overall survival was 34.0 months (not reached at RP2D), with an estimated 2-year overall survival of 87.4%. The commonest Grade ≥3 AEs included keratopathy (52.6%), decreased visual acuity (39.5%), neutropenia (36.8%), and thrombocytopenia (34.2%), leading to dose reductions in 63.2% of patients but only 5.2% discontinuing treatment due to AEs. Notably, no irreversible vision loss was reported, and extended dosing intervals at 2.5 mg/kg Q8W reduced ocular toxicity while maintaining efficacy, supporting its evaluation in the ongoing Phase III DREAMM-8 trial, comparing belantamab mafodotin plus pomalidomide and dexamethasone vs. bortezomib plus pomalidomide and dexamethasone as a standard-of-care regimen for RRMM patients [21].

Additionally, in 2022, Abeykoon et al. [30], published a retrospective study about ocular toxicity among patients with RRMM who underwent treatment with a dose of belantamab mafodotin at 2.5 mg/kg. In total, 38 patients were included, and 36 completed with a median follow-up of 11 months; the ORR was 29% with the median response duration being three months, the median PFS two months, and the overall survival rate was between 40% and 50%. Three patients had VGPR. Nearly 75% of patients had ocular toxicity, with 69% developing keratopathy. Due to keratopathy, nine patients had treatment delay, and four needed dose reduction. Moreover, five patients discontinued treatment, with two fatalities [30].

In the same year, the Spanish Expanded Access Program (EAP) study was published by Alegre et al. [31], which assessed real-world outcomes of belantamab mafodotin in RRMM patients. Conducted at 14 hospitals in Madrid, Spain, this observational, retrospective, multicenter study included 33 patients (median age: 70 years, range 46–79) who had received a median of 5 prior therapy lines (range: 3–8), with 90% classified as triple-, quad-, or penta-refractory. Patients received belantamab mafodotin (2.5 mg/kg IV every 3 weeks). After a median follow-up of 11 months (range: 6–15 months), the ORR was 42.2%, with 18.2% achieving ≥VGPR. The median PFS in the total cohort was 3 months (95% CI: 0.92–5.08) but increased to 11 months in patients who achieved ≥PR. The overall median survival duration was 13 months (95% CI: 3.6–24.7 months). Non-hematologic AEs [17] occurred in 57.6% of patients (30.3% Grade ≥3), with keratopathy (51.5%) and patient-reported vision-related symptoms (45.5%) being the most common. Keratopathy resolved in 70.6% of patients. Grade ≥3 hematologic AEs (18.2%) included thrombocytopenia (21.2%). Dose reductions (30.3%), dose delays (36.4%), and treatment discontinuation (54.5% due to progression, 15.1% due to AEs) were observed. Despite ocular toxicity concerns, the study confirmed belantamab mafodotin’s efficacy and manageable safety profile, aligning with findings from DREAMM-2 [31].

Then, in 2023, the Spanish Compassionate Use and Expanded Access Program (EAP) study was published by de la Rubia et al. [28]. In this study, the researchers analyzed the real-world data on belantamab mafodotin monotherapy in 156 RRMM patients treated across 84 Spanish hospitals between November 2019 and June 2021. Patients had received a median of 5 prior therapy lines (range 1–10), with 88% being triple-class refractory and 34.6% penta-refractory. They were followed up for a median of 10.9 months (range 1–28.6 months), the ORR was 41.8%, with ≥CR in 13.5%, VGPR in 9%, PR in 17.3%, and MR in 2%. The median PFS increased from 3.61 months (95% CI: 2.1–5.1) to 14.47 months (95% CI: 7.91–21.04) among patients who were achieving MR or better (*p* < 0.001). Median overall survival was 11.05 months (95% CI: 8.7–13.3) and 23.35 months in responders (≥MR). Corneal events (87.9%; Grade ≥3 in 33.7%) were the most common AEs, with thrombocytopenia (15.4%) and infections (15%) also observed. Despite ocular toxicity, only 2 patients (1.3%) discontinued treatment due to side effects. These findings support belantamab mafodotin’s real-world efficacy and safety in heavily pretreated RRMM patients, consistent with the results from DREAMM-2 results [28].

In 2024, the Italian retro-prospective observational study was published by Fazio et al., which evaluated the real-world efficacy and safety of belantamab mafodotin in triple-class refractory multiple myeloma (TCR-MM) patients treated under named patient and expanded access programs from 2020 to 2022. Conducted at 34 Italian centers, the study included 78 patients (median age 65 years, range 42–86), who had received a minimum of four prior lines of therapy, with 64% receiving five or more. A disease control rate of 49% was observed (36/74 evaluable patients), with 43% achieving at least a PR, 28% a VGPR, and 13.5% a CR. The patients were followed up for a median of 12 months (range 6–21 months) with the median DOR being 14 months, the median PFS 5.5 months, and the median overall survival 12 months. Age >70 years, good performance status, and achieving at least MR were associated with longer PFS and overall survival. Ocular toxicity was the most common AE, with keratopathy occurring in 58% of patients (Grade 3 in 2.5%), corneal symptoms in 32% (Grade 3 in 1.2%), and reduced visual acuity in 14%, but only 3% discontinued treatment due to AEs. Grade 3 thrombocytopenia was observed in 9% of patients. This real-world study confirms the efficacy and durability of belantamab mafodotin in TCR-MM patients, particularly those ineligible for novel immunotherapies like chimeric antigen receptor T or bispecific antibodies, supporting its continued use with careful ocular toxicity management [26].

In 2024, the BelaRd Phase I/II study (NCT04808037) was published by Terpos et al. This study evaluated the safety and efficacy of belantamab mafodotin plus lenalidomide and dexamethasone in multiple myeloma patients who were newly diagnosed but who were ineligible for autologous stem cell transplantation. Conducted in Greece by the Hellenic Society of Haematology, it enrolled 36 elderly patients (median age: 72.5 years, range: 64–86) randomized 1:1:1 to receive belantamab mafodotin at 2.5, 1.9 , or 1.4 mg/kg every 8 weeks (Q8W), later extended to every 12 weeks (Q12W) to mitigate ocular toxicity. The patients were followed-up for a median of 20.3 months achieving 100% ORR, with 83.3% achieving ≥VGPR, and 52.8% achieving ≥CR, while MRD negativity was achieved in 73.7% of patients with ≥CR. The recommended Phase II dose (RP2D) was determined to be 1.9 mg/kg Q8W, later adjusted to Q12W to balance efficacy and safety. Ocular toxicities were dose-dependent, with Grade ≥3 keratopathy rates of 4.2% (2.5 mg/kg cohort), 0.4% (1.9 mg/kg), and 0.5% (1.4 mg/kg), leading to dose withholding in 66.7%, 91.7%, and 100% of patients, respectively, though median resolution time was 1.0–1.4 months. The commonest Grade ≥3 AEs included fatigue (58.3%), diarrhea (22.2%), thrombocytopenia (33.3%), and leukopenia (41.7%), with six infection-related deaths (four due to COVID-19, two from pneumonia). The study concluded that belantamab mafodotin plus lenalidomide and dexamethasone achieves deep and durable responses in transplant-ineligible newly diagnosed multiple myeloma patients, with no disease progression at 20.3 months, supporting its potential for further evaluation in Phase II/III trials [27].

MEDI2228 is a BCMA-targeted ADC conjugated with the pyrrolobenzodiazepine dimer tesirine. In 2024, Dimopoulos and colleagues published the findings of the Phase I first-in-human study of MEDI2228 (NCT03489525). The researchers evaluated the safety, tolerability, and efficacy of MEDI2228 in RRMM patients. Conducted across 10 sites in the United States, Australia, and Greece, it had 107 participants with a median of five prior lines of treatment (range: 2–17); all were previously treated with immunomodulatory drugs, PIs, and anti-CD38 monoclonal antibodies. The maximum tolerated dose (MTD) was established at 0.14 mg/kg every 3 weeks (Q3W), with an additional dose once every 6 weeks (Q6W) expansion cohort. The ORR was 39.3% across all doses, 56.1% at MTD, and 53.3% in triple-refractory patients, with a median PFS of 6.6 months (MTD) and 5.1 months (Q6W). Median overall survival was 12.9 months (MTD) and 15.8 months (Q6W), with a DOR of 5.9 months. The most common AEs [17] included photophobia (43.9%), rash (29.0%), thrombocytopenia (19.6%), and fatigue (44.9%), while Grade ≥3 AEs included thrombocytopenia (36.6%), gamma-glutamyl transferase increase (26.8%), and photophobia (17.1%). Ocular toxicity, particularly photophobia, affected 57% of patients, leading to 14% discontinuation but without keratopathy or visual acuity loss. Ultimately, frequent treatment discontinuations due to ocular toxicity precluded further clinical development of MEDI2228, underscoring the need for improved payloads and dosing strategies for BCMA-targeted ADCs [22].

AMG 224 is a BCMA-ADC consisting of a BCMA IgG1 antibody conjugated, through a non-cleavable linker, with mertansine. In 2021, Lee et al. assessed the maximum dose safety and tolerability, and efficacy of AMG 224 in relapse/refractory patients. Twenty-one RRMM patients were recruited in the dose escalation group (30–250 mg) and 11 in the dose expansion group (3 mg/kg). The total overall response rate was 23% (9/40), with six of 29 patients in the group that had dose escalation and three of 11 in the group that had dose expansion. Twenty-one patients developed treatment-related SAEs: 16 in the dose escalation group and five in the dose expansion group. The most common AEs were thrombocytopenia, neutropenia, and anemia. Due to these, one patient required a dose reduction and three discontinued treatments. Additionally, around 30% of patients had ocular AEs. However, no dose reduction, or treatment delay/discontinuation was required due to ocular toxicity [32].

### 3.2 Animal Studies

Five animal studies were included in the review, relating to belantamab mafodotin, MEDI2228, and HDP-101 (see Table S2).

In 2014, Tai et al. [38] published a study involving 40 xenograft mice to explore the effect of belantamab mafodotin on multiple myeloma. The results showed that the group treated with a dose of 4 mg/kg of belantamab mafodotin had complete tumor eradication during the study, and the tumor-free days were up to three months. No AE was observed [38]. In 2021, de Oca et al. [39] published a study revealing a novel immune-mediated mechanism of action of belantamab mafodotin against lymphoma. The results showed that the combination of belantamab mafodotin and a mouse anti-OX40 IgG2a antibody could inhibit lymphoma growth in an animal model. No AE was reported [39].

MEDI2228 is an ADC containing BCMA antibody and a highly cytotoxic DNA minor-groove interstrand-crosslinking pyrrolobenzodiazepine dimer. In 2020, Xing et al. [40] reported the anti-cancer effects of MEDI2228 combined with bortezomib in a xenograft mouse model. The results showed 0.4 mg/kg of MEDI2228 combined with bortezomib had the strongest anti-cancer effect against multiple myeloma, with no AE [40]. In 2021, Xing et al. [41] published another study about the effect of MEDI2228 combined with human natural killer (NK) cells and daratumumab against multiple myeloma in a xenograft mouse lacking NK cells. The dose of MEDI2228 was 0.3 mg/kg, which was considered to be the optimal dose for this combination. All mice in this group survived, and the tumor-free days extended to 116 days. No AE was found in this group [41].

HDP-101 is a BCMA-ADC conjugated with an amanitin derivative. In 2021, Figueroa-Vazquez et al. [42] published a study on cytotoxic potency and tolerability of HDP-101 against multiple myeloma in a xenograft mouse and cynomolgus monkey model. The results showed HDP-101 could induce tumor regression at a low dose, such as 2.2 mg/kg. The highest survival rate was 100% in the mouse group, and the longest tumor-free days were more than 100, which demonstrated a promising therapeutic effect of this ADC. No AE was found in the mouse group. In the cynomolgus monkey group, the liver and kidneys were affected by adverse effects. In the liver, transient elevations of liver biomarkers were found, while in the kidneys, signs of toxicity, including interstitial nephritis, hyaline tubular casts, cortical fibrosis, and necrosis of tubular cells were noted, although these symptoms demonstrated partial or complete recovery after a treatment-free period [42].

### 3.3 Quality Assessment

The ROBINS-I tool was used to assess the quality and risk of bias of the human clinical studies. Common issues included lack of blinding (participants and assessors), small sample sizes, non-randomized designs, and inconsistent AE reporting. Four studies reported low risk of confounding, and ten studies showed low risk of participant selection bias. Five trials had a low risk of classification of interventions, and six trials had a low risk of deviations from intended interventions. Six studies had a low risk of missing data. Two trials had a low risk of outcome measurement. Only two studies showed a low risk of bias from selective reporting of results. In total, based on the ROBINS-I criteria, five studies were assessed as having a serious risk of bias, nine studies as having a moderate risk of bias, and two studies as having a low risk of bias (Table 2).

**Table 2:** ROBINS-I for quality assessment

Bias due to confounding	Bias in selection of participants	Bias in classification of interventions	Bias due to deviations from intended interventions	Bias due to missing data	Bias in measurement of outcomes	Bias in selection of reported results	Overall risk of bias	Author	Year of publication
Moderate	Low	Moderate	Moderate	Low	Moderate	Moderate	Moderate	Trudel et al. [21]	2024
Moderate	Low	Moderate	Moderate	Moderate	Moderate	Moderate	Moderate	Dimopoulos et al. [22]	2024
Low	Low	Low	Low	Low	Moderate	Low	Low	Popat et al. [23]	2024
Moderate	Low	Moderate	Moderate	Moderate	Moderate	Moderate	Moderate	Suvannasankha et al. [24]	2024
Moderate	Low	Moderate	Moderate	Moderate	Moderate	Moderate	Moderate	Dimopoulos et al. [25]	2023
Moderate	Low	Moderate	Moderate	Moderate	Moderate	Moderate	Moderate	Fazio et al. [26]	2024
Low	Low	Low	Low	Low	Moderate	Low	Low	Terpos et al. [27]	2024
Moderate	Low	Moderate	Moderate	Moderate	Moderate	Moderate	Moderate	de la Rubia et al. [28]	2023
Moderate	Low	Moderate	Moderate	Moderate	Moderate	Moderate	Moderate	Nooka et al. [29]	2023
Moderate	Moderate	Low	Low	Moderate	Serious	Low	Serious	Abeykoon et al. [30]	2022
Moderate	Low	Moderate	Moderate	Moderate	Moderate	Moderate	Moderate	Alegre et al. [31]	2023
Moderate	Moderate	Low	Low	Low	Low	Serious	Serious	Lee et al. [32]	2021
Low	Moderate	Low	Low	Low	Low	Moderate	Serious	Lonial et al. [33]	2021
Moderate	Low	Serious	Serious	Low	Moderate	Moderate	Serious	Richardson et al. [34]	2020
Moderate	Moderate	Moderate	Moderate	Low	Moderate	Serious	Serious	Trudel et al. [35]	2019
Moderate	Low	Low	Moderate	Moderate	Low	Moderate	Moderate	Trudel et al. [21]	2018

Note: **Low**, any bias is unlikely to change the conclusions; **Moderate**, bias may lower confidence but not overturn results; **Serious,** important problems likely to substantially distort the effect estimate.

The SYRCLE risk of bias tool was used to assess the quality of the animal studies. All five studies used generations of a sequence of random numbers. Two trials blinded their outcome assessments, and another two studies blinded their outcome assessors. All five studies show low risk of incomplete outcome data and selective outcome reporting (Table 3).

**Table 3:** SYRCLE’s risk of bias assessment

Random sequence generation	Baseline characteristics	Allocation concealment	Random housing	Blinding	Random outcome assessment	Blinding	Incomplete outcome data	Selective outcome reporting	Author	Year of publication
Selection bias	Selection bias	Selection bias	Performance bias	Performance bias	Detection bias	Detection bias	Attrition bias	Reporting bias		
Low	High	High	Unknown	High	Low	High	Low	Low	Figueroa-Vazquez et al. [42]	2021
Low	High	High	Unknown	High	High	High	Low	Low	de Oca RM et al. [39]	2021
Low	Unknown	Unknown	Unknown	Unknown	Unknown	Low	Low	Low	Xing et al. [41]	2021
Low	Unknown	Unknown	Unknown	Unknown	Unknown	Low	Low	Low	Xing et al. [40]	2020
Low	Unknown	High	Unknown	High	Low	High	Low	Low	Tai et al. [38]	2014

Note: Low, the study provides sufficient information to indicate the bias is unlikely to influence the outcome; High, the study provides sufficient information to suggest the bias is likely to influence the outcome; Unknown, Insufficient information is provided to determine the risk of bias for that domain.

The quality assessment tool mentioned by Chou et al. [43], for AEs was used only for human clinical studies, due to the lack of an appropriate equivalent tool for animal studies. No significant bias was detected in the domains of selection, loss to follow-up, AEs being pre-specified and defined, adequately describing ascertainment technique, ascertainment of AEs, and duration of follow-up [43]. One study, however, did not adequately provide a description of the population [30]. None of the studies adequately showed statistical analysis of potential confounders. Therefore, the overall ranking for this outcome for 16 studies was considered as ‘good’ [32–36], and for the remaining study was ‘fair’ [30] (Table 4).

**Table 4:** Quality assessment for adverse events via the tool by Chou et al

NBS	ADP	LLFU	AEP	ATD	NBA	ASA	AFU	TS	QC	Author	Year of publication
1	1	1	1	1	1	1	1	8	Good	Trudel et al. [21]	2024
1	1	1	1	1	1	1	1	8	Good	Dimopoulos et al. [22]	2024
1	1	1	1	1	1	1	1	8	Good	Popat et al. [23]	2024
1	1	1	1	1	1	1	1	8	Good	Suvannasankha et al. [24]	2024
1	1	1	1	1	1	1	1	8	Good	Dimopoulos et al. [25]	2023
1	1	1	1	1	1	1	1	8	Good	Fazio et al. [26]	2024
1	1	1	1	1	1	1	1	8	Good	Terpos et al. [27]	2024
1	1	1	1	1	1	1	1	8	Good	de la Rubia et al. [28]	2023
1	1	1	1	1	1	1	1	8	Good	Nooka et al. [29]	2023
1	1	1	1	1	1	1	1	8	Good	Abeykoon et al. [32]	2022
1	0	1	1	1	1	0	1	6	Fair	Alegre et al. [31]	2023
1	1	1	1	1	0	1	1	7	Good	Lee et al. [32]	2021
1	1	1	1	1	1	0	1	7	Good	Lonial et al. [33]	2021
1	1	1	1	1	1	0	1	7	Good	Richardson et al. [34]	2020
1	1	1	1	1	1	0	1	7	Good	Trudel et al. [35]	2019
1	1	1	1	1	1	0	1	7	Good	Trudel et al. [21]	2024

Note: NBS, Non-Biased Selection; ADP, Adequate Description of Population; LLFU, Low Loss to Follow-Up; AEs, Adverse Events Pre-Specified; ATD, Ascertainment Technique Described; NBA, Non-Biased Ascertainment; ASA, Adequate Statistical Analysis; AFU, Adequate Follow-Up; TS, Total of Score; QC, Quality Classification. >6: Good: “These are studies that fulfill most or all of the quality criteria. They have negligible flaws, and their results are considered reliable.” 4–6: Fair: “These are studies that fulfill several but not all of the quality criteria. While they have some flaws, they do not fatally undermine the study. The results are likely valid, but caution should be exercised in their interpretation.” <4: Poor: “These are studies that have significant flaws that might invalidate the results. They fulfill a few quality criteria, and caution is advised when interpreting these studies.”.

## 4 Discussion

This systematic review analyzed 21 studies that investigated the use of BCMA-ADCs, primarily belantamab mafodotin, AMG 224, MEDI2228, and HDP-101, for the treatment of multiple myeloma, with 16 human studies and five animal studies included.

For human studies, belantamab mafodotin, a BCMA-targeted ADC, has demonstrated durable responses in RRMM across multiple clinical trials, including the DREAMM series, with the ORR ranging from 32% to 85% and PFS from 2.8 to 36.6 months. However, its widespread use is limited by ocular toxicity (keratopathy, blurred vision), requiring frequent dose modifications. Combination regimens (DREAMM-6, DREAMM-7, DREAMM-8) have improved efficacy while modifying dosing strategies to mitigate toxicity. Real-world studies confirm its effectiveness but emphasize careful ocular management. MEDI2228 demonstrated promising efficacy (ORR 39.3%, PFS 6.6 months) but was associated with significant ocular toxicity, particularly photophobia, suggesting the need for optimized payloads or dosing strategies to improve tolerability. AMG 224 (ORR 23%) showed a favorable pharmacokinetic profile and manageable toxicity but was linked to high rates of thrombocytopenia, indicating that future modifications could focus on reducing hematologic AEs while maintaining efficacy.

All of the recently conducted clinical trials involving ADC are related to the treatment of multiple myeloma, and mostly explored the use of belantamab mafodotin. Bortezomib plus melphalan and prednisone is empirically used as an initial treatment for multiple myeloma. A real-life clinical study showed that the regimen’s completion rate was around 10% with a median survival of about three years [44]. belantamab mafodotin is used to treat RRMM patients who fail to respond to their first-line treatment. Another FDA-approved antimyeloma agent is daratumumab (Darzalex), which is the first-in-class human-specific anti-CD38 IgG1 monoclonal antibody [45]. However, due to the pleiotropic effects of drug deregulation, daratumumab resistance happens in clinical practice [46], which has stimulated a search for novel treatment strategies, such as ADC, bispecific antibody, and chimeric antigen receptor T cell regimens. Most of these are targeting RRMM [47], but unlike chimeric antigen receptor T and bispecific antibody, ADCs do not depend on T-cell activity, and no life-threatening T-cell-related cytokine release syndrome has been found in ADC treatment.

We are aware of some conference abstracts on additional studies from the DREAMM trial series without full reports or results. DREAMM-5 (NCT04126200) is an ongoing Phase I/II platform trial designed to evaluate belantamab mafodotin combined with novel agents for RRMM. The study explores four combination regimens: belantamab mafodotin plus an OX40 agonist (GSK3174998), an inducible T-cell costimulator (ICOS) agonist (feladilimab, GSK3359609), a gamma-secretase inhibitor (nirogacestat), or a PD-1 inhibitor (dostarlimab), compared to belantamab mafodotin monotherapy. The trial follows a platform study design, allowing for continuous evaluation and adaptation based on emerging data. The primary endpoints include safety, tolerability, and ORR, with secondary assessments on PFS, DOR, and MRD negativity. The study aims to identify effective belantamab mafodotin-based combinations that enhance efficacy while minimizing toxicity, particularly addressing ocular AEs. By incorporating a master protocol with adaptive sub-study designs, DREAMM-5 seeks to streamline the development of synergistic treatments for RRMM [48]. The DREAMM-9 (NCT04091126) Phase I trial is an ongoing open-label, randomized study evaluating the safety, efficacy, and pharmacokinetics of belantamab mafodotin combined with bortezomib, dexamethasone, and lenalidomide (VRd) among newly diagnosed multiple myeloma patients who were not eligible for transplant. In a study of 36 patients (median age 74), the interim data showed promising efficacy and safety across all cohorts, with over 50% achieving VGPR or better and several CRs. These preliminary findings suggest that belantamab mafodotin plus VRd demonstrates high response rates with a manageable safety profile in newly diagnosed multiple myeloma patients, though longer follow-up is required to confirm its long-term efficacy and tolerability [49].

Although formal resistance analyses were not reported across included trials, reduced membrane BCMA density, for example via γ-secretase–mediated shedding with sequestration as soluble BCMA, could limit ADC target engagement; accordingly, DREAMM-5 is prospectively testing nirogacestat (γ-secretase inhibitor) to increase membrane BCMA and enhance belantamab activity. Combination regimens have shown the most consistent efficacy gains: belantamab mafodotin improved median PFS to 36.6 months with an ORR of 83% vs. 13.4 months for non-combination regimens in DREAMM-7, albeit with higher ocular events; belantamab + lenalidomide/dexamethasone achieved an ORR of 67% and a median PFS of 18.4 months in DREAMM-6; and belantamab + pomalidomide/dexamethasone reached an ORR of 85.3% at RP2D on an every-8-weeks schedule (DREAMM-8). In contrast, the pembrolizumab combination (DREAMM-4) showed activity but no substantial durability advantage vs. monotherapy, and DREAMM-3 highlighted the limitation of single-agent belantamab, reinforcing the rationale for rational combinations and schedule optimization (e.g., Q8W). Together, these data support target-density strategies (e.g., γ-secretase inhibition) and PI/IMiD-based combinations as near-term avenues to improve efficacy while managing ocular toxicity. As described in our results, ocular toxicity is the most common AE associated with the use of BCMA-ADCs, including belantamab mafodotin and MEDI2228. The mechanism of ocular toxicity is still unclear. The ADC-related ocular toxicity may be caused by an on-target or off-target process. The on-target mechanism is where the non-cancerous cells express the target antigen, binding to the mAb; therefore, the payload can be released into these cells. Alternatively, the off-target mechanism is the non-cancerous cells without target antigen expression that are damaged by the payload. These may relate to Fc-receptor-mediated endocytosis, pinocytosis, and bystander cytotoxicity [50,51]. Ocular toxicity from belantamab mafodotin is thought to arise either via drug entry through the vascularized limbus or accumulation in the tear film, supported by observations of peripheral-to-central corneal lesions and the presence of drug payload in rabbit and patient tear samples [52,53].

Although the underlying mechanism is not fully understood, converging clinical/experimental data support a predominantly off-target model in which corneal epithelial cells take up intact ADC or released payload via Fc-receptor–mediated endocytosis and macropinocytosis, with bystander cytotoxicity contributing to epithelial injury. Two plausible exposure routes have been described for belantamab mafodotin: trans-limbal entry from the vascularised limbus and accumulation in the tear film, which is consistent with the peripheral-to-central lesion pattern and detection of drug-related material in rabbit and patient tear samples. Payload chemistry appears to shape the phenotype: MMAF-conjugates are associated with keratopathy and reduced visual acuity, while the PBD-based MEDI2228 produced photophobia without keratopathy/visual-acuity loss (any-grade ~57%; 14% discontinuations for ocular symptoms). Mitigation centers on baseline/serial ophthalmic assessments, use of preservative-free lubricants, and dose holds/reductions; longer dosing intervals (e.g., 2.5 mg/kg Q8W) and, in selected settings, every 12 weeks (Q12W), have been associated with lower ocular-event rates while maintaining efficacy [54]. Future clinical studies should prioritize comprehensive reporting of exposure-toxicity relationships to facilitate deeper quantitative assessments and enhance toxicity management strategies.

The other most common SAEs are hematological toxicity, especially regarding the use of AMG 224, including thrombocytopenia, neutropenia, and anemia. These also caused dose reduction, treatment delay, and treatment discontinuation in clinical trials. Generally, these AEs may relate to two mechanisms: (i) a decreased medullary production of progenitor cells and precursors caused by the payload cytotoxicity on megakaryocytes and myeloblasts; (ii) an immune-mediated effect on platelet and neutrophil destruction in peripheral blood [55]. Although several ADC studies have been conducted to minimize these effects, an ideal solution has not yet been achieved. Further study in this field is also required.

Animal studies evaluating belantamab mafodotin, MEDI2228, and HDP-101 in xenograft mouse models showed promising anti-cancer effects and tolerability. Belantamab mafodotin has been tested against lymphoma in an animal study, and the results were encouraging, suggesting a wider possible use of belantamab mafodotin in malignant disease treatment. Additionally, several new BCMA-ADCs against multiple myeloma have been developed with different payloads and linkers, whose results were also promising *in vivo* studies. These may provide a chance to maximize the payload delivery to tumor tissue and minimize the delivery to normal tissues, for achieving optimal efficacy with fewer AEs. The discrepancy between preclinical and clinical data, especially in terms of toxicity, underscores the limitations of animal models in fully predicting human responses. This gap arises from interspecies differences and oversimplified preclinical conditions; thus, integrating advanced *in vitro* systems, humanized models, and computational approaches may improve translational accuracy and reduce clinical risks.

The quality assessment of the studies included in this systematic review is an essential aspect to consider when discussing the findings and drawing conclusions. The review employed two well-established tools to evaluate the risk of bias and quality of the studies: ROBINS-I for human clinical studies and SYRCLE’s risk of bias tool for animal studies. For the human clinical studies, the ROBINS-I assessment revealed that five studies had a serious risk of bias. Although these biases may impact the interpretation of the results, it is important to note that the majority of the studies included were early-phase trials (phase I or II), which inherently have limitations in terms of sample size and generalizability. Additionally, the studies varied in design and treatment regimens, further contributing to the heterogeneity and potential biases. However, the presence of biases does not necessarily negate the value of the studies, as they still provide valuable information on the safety and efficacy of BCMA-ADCs for RRMM patients. The findings from these studies can serve as a foundation for future research, which should aim to address the identified biases through larger, phase III trials and more standardized designs.

The animal studies were assessed using SYRCLE’s risk of bias tool, and all five studies demonstrated low risk of incomplete outcome data and selective outcome reporting. However, not all studies blinded their outcome assessments or outcome assessors, which may introduce bias into the results. Despite these potential biases, the animal studies provide crucial insights into the mechanisms of action, safety, and efficacy of BCMA-ADCs in preclinical settings. These findings can guide the development of future clinical trials and contribute to the optimization of BCMA-ADC therapies for RRMM patients.

While the majority of clinical data currently available centers around belantamab mafodotin, primarily due to its advanced clinical development and the availability of published studies, our interpretation is not based solely on its performance. Rather, this emphasis reflects the current distribution of available evidence and does not indicate methodological bias. Consistent cross-programme themes for BCMA-ADCs include resistance via antigen modulation—ranging from therapy-associated BCMA loss in a subset of patients to γ-secretase–mediated shedding that lowers cell-surface target density and raises soluble BCMA—with pharmacologic γ-secretase inhibition increasing membrane BCMA and potentially restoring sensitivity [56]. In parallel, combination strategies have produced clinically meaningful improvements, with belantamab-based triplets alongside immunomodulatory imide drugs (IMiDs) or PIs prolonging progression-free and overall survival. Furthermore, the efficacy signals observed in preclinical animal studies of novel BCMA-targeting ADC candidates may overcome the limitations of earlier agents. Although agents like MEDI2228 and AMG224 have limited clinical data, their reported clinical outcomes nevertheless contribute additional support to the therapeutic rationale for BCMA-targeting ADCs. Taken together, the mechanistic foundation, early clinical signals, and encouraging animal data support the need for further research into the broader potential of BCMA-ADCs across various malignancies.

## 5 Strengths and Limitations

A strength of this review is that it used a systematic search strategy to identify relevant studies on the development of BCMA-ADCs. This approach minimized the risk of missing important research articles in the field. Including both human clinical trials and animal studies, it provided a broader understanding of the safety and efficacy of BCMA-ADCs in various settings and stages of development. The review employed robust tools to assess the quality of included studies, such as ROBINS-I for human clinical studies and SYRCLE’s risk of bias tool for animal studies.

This systematic review has several limitations. First, because of substantial clinical heterogeneity across studies, a meta-analysis could not be conducted. Second, as specified in our PROSPERO protocol, we did not perform direct comparisons with other BCMA-targeted modalities such as CAR-T cell therapies or bispecific antibodies. Third, the certainty of the available evidence is constrained by the predominance of early-phase, often single-arm trials with small sample sizes and limited follow-up, which increases imprecision and susceptibility to bias. The included studies enrolled narrowly defined populations and exhibited substantial variation in prior treatments, dosing regimens, supportive care practices, and outcome ascertainment windows, limiting cross-study comparability and external validity. Adverse event definitions, grading systems, and monitoring frequency—especially for ocular toxicities—were not uniform, raising concerns about differential or incomplete harms reporting. Finally, given the small number of studies, the potential for reporting or publication bias cannot be excluded.

## 6 Conclusion

BCMA is an ideal antigen for multiple myeloma-specific immunotherapeutic, and ADC is an ideal target therapy to reduce the treatment related impact on normal tissues. ADCs represent a rational approach to selectively deliver cytotoxic agents while sparing normal tissues. Clinical trials of BCMA-targeting ADCs—including belantamab mafodotin, MEDI2228, and AMG 224—have demonstrated anticancer activity, though ocular and haematologic toxicities remain challenges. Preclinical studies of newer candidates support continued development in this drug class. Future studies should focus on enhancing efficacy and reducing toxicity across diverse treatment settings.

## Supplementary Materials

Supplementary Table S1 Human clinical study

Supplementary Table S2 Animal study

Supplementary Table S3 Comparative summary of BCMA-targeted ADCs

Supplementary Material S4 Ovid MEDLINE search strategy

Supplementary Material S5 PRISMA checklist

## Data Availability

The data that support the findings of this study are available from the corresponding author upon reasonable request.

## Supplementary Materials

The supplementary material is available online at https://www.techscience.com/doi/10.32604/or.2025.070851/s1. Supplementary Table S1: Human clinical study; Supplementary Table S2: Animal study; Supplementary Table S3: Comparative summary of BCMA-targeted ADCs; Supplementary Material S4: Ovid MEDLINE search strategy; Supplementary Material S5: PRISMA checklist.
