# Supplementary material for: The Efficacy and Safety of B-Cell Maturation Antigen (BCMA) Antibody-Drug Conjugates (ADC) in Development against Cancer: A Systematic Review
Source: Oncol Res. 2025 Dec 30;34(1):1. doi: 10.32604/or.2025.070851 (PMC12774553; doi:10.32604/or.2025.070851)
Supplement: Supplementary Table S3 [file OncolRes-34-70851-s003.docx]

Table S3. Comparative summary of BCMA-targeted ADCs

| Payload class | Molar‑equivalent dosing and safety | Antigen density / BCMA expression | Clinical outcome (key trials) | Comparative insights/trends |
| --- | --- | --- | --- | --- |
| Microtubule inhibitor (auristatin F) | Dosed by mg/kg across studies (e.g., 2.5 or 3.4 mg/kg Q3W; combinations include 2.5 mg/kg Q3W and 2.5 mg/kg Q8W). Exposure was not normalized across programs (DAR/exposure not consistently reported). DLTs: none identified in Phase I dose‑escalation; no DLTs reported in DREAMM‑6. | BCMA surface density is a key determinant of internalization; increasing membrane BCMA via γ-secretase inhibition is being tested in DREAMM‑5 to enhance engagement. | Monotherapy: ORR 32–60%, median PFS 2.8–12.0 mo (DREAMM‑1/2); Final DREAMM‑2: ORR 32–35%, PFS 2.8–3.9 mo, frequent dose modifications. Combinations: BVd (DREAMM‑7) ORR 83%, PFS 36.6 mo vs 13.4 mo; Bela + Len/Dex (DREAMM‑6) ORR 67%, PFS 18.4 mo; Bela + Pom/Dex (DREAMM‑8) ORR 85.3%, PFS not reached at RP2D (Q8W). Major AEs: keratopathy ~51–96% (Grade ≥3 ~18–53%), Grade ≥3 thrombocytopenia 22–57%, anaemia 16–28%. | Strongest evidence base: ocular toxicity (keratopathy) is class-defining for MMAF ADCs; longer intervals (Q8W/Q12W) can mitigate ocular events while maintaining activity; PI/IMiD backbones yield the largest efficacy gains vs monotherapy. |
| DNA-damaging PBD dimer (tesirine) | 0.14 mg/kg Q3W MTD; additional Q6W cohort. Exposure normalization not reported; safety dominated by ocular symptoms and cytopenias. | Target‑engagement assumed; specific BCMA density reporting was not consistent in the Phase I publication. | Phase I: ORR 39.3% overall (56.1% at MTD); median PFS 5.1–6.6 mo; discontinuation for ocular symptoms 14%. Major AEs: photophobia 43.9% (Grade ≥3 17.1%), Grade ≥3 thrombocytopenia 36.6%, ↑GGT 26.8%; no keratopathy/VA loss reported. | A distinct ocular phenotype (photophobia without keratopathy) suggests payload‑dependent toxicity; frequent ocular discontinuations curtailed development. |
| Microtubule inhibitor (maytansinoid) | Dosed as mg (30–250 mg) in escalation; 3 mg/kg in expansion. Exposure normalization not reported; cytopenias prominent. | BCMA expression not systematically reported; internalization presumed. | Phase I: ORR 23% (9/40). Major AEs: Grade 4 thrombocytopenia 24%, anaemia 21%, ocular AEs 21%; PFS not consistently reported. | Lower activity signal vs other programs with hematologic toxicity prominent; ocular AEs present but less defining than with MMAF. |
| Amanitin-based transcription inhibitor (preclinical) | Preclinical dosing (e.g., 2.2 mg/kg in mice). Monkey tox: reversible hepatic enzyme rises; renal changes (interstitial nephritis, casts, fibrosis/necrosis) with recovery after washout. | Antigen density not quantified; robust preclinical activity despite low‑proliferation states reported in the study. | Mice: tumour regression and >100 tumour-free days at low doses; Monkeys: liver/kidney findings with partial/complete recovery off‑treatment. No human efficacy data yet. | Mechanistically distinct payload with non-overlapping ocular profile (preclinical); translational safety (hepatic/renal) is the key risk to watch in first-in-human studies. |

Note: mc, maleimidocaproyl; MMAF, monomethyl auristatin F; PBD, pyrrolobenzodiazepine; DM1, mertansine; Val‑Ala, valine–alanine; BVd, belantamab mafodotin + bortezomib + dexamethasone; Len/Dex, lenalidomide/dexamethasone; Pom/Dex , pomalidomide/dexamethasone; ORR, overall response rate; PFS, progression-free survival; DLT, dose-limiting toxicity; RP2D, recommended Phase II dose; VA, visual acuity; GGT, increased gamma-glutamyl transferase.

**Supplementary Material 4: Ovid MEDLINE search strategy**

Database: Ovid MEDLINE(R) ALL, 1946 -

Search Strategy:

--------------------------------------------------------------------------------

1 exp B-Cell Maturation Antigen/

2 ("b cell" adj2 matur$ adj2 antigen$).tw.

3 bcma$.tw.

4 ("b cell" adj2 matur$ adj2 protein$).tw

5 (cluster adj2 differentiat$ adj2 "269").tw.

6 ("CD 269" or CD-269 or CD269).tw.

7 exp Receptors, Tumor Necrosis Factor/

8 "tumour necrosis factor receptor superfamily member 17".tw.

9 "tumor necrosis factor receptor superfamily member 17".tw.

10 ("TNFRSF 17" or TNFRSF-17 or TNFRSF17).tw.

11 1 or 2 or 3 or 4 or 5 or 6 or 7 or 8 or 9 or 10

12 exp Immunoconjugates/

13 ((antibody adj2 drug$ adj2 conjugate$) or (antibody-drug$ adj2 conjugate$)).tw.

14 ADC$.tw.

15 12 or 13 or 14

16 11 and 15

17 exp Treatment Outcome/

18 efficac$.tw.

19 effective$.tw.

20 exp Safety/

21 exp Safety-Based Drug Withdrawals/

22 exp Product Surveillance, Postmarketing/

23 exp Drug Evaluation/

24 exp Adverse Drug Reaction Reporting Systems/

25 (adverse adj2 (effect$ or event$)).tw.

26 (safe or safety).tw.

27 reactogenic$.tw.

28 ((postmarketing or post-marketing or post marketing or postlicensure or post-licensure or post licensure) adj5 (surveillance or monitor$)).tw.

29 pharmacovigilan$.tw.

30 17 or 18 or 19 or 20 or 21 or 22 or 23 or 24 or 25 or 26 or 27 or 28 or 29

31 16 and 30
